# How musical engagement promotes well-being in education contexts: The case of a young man with profound and multiple disabilities

**DOI:** 10.3402/qhw.v8i0.20570

**Published:** 2013-08-07

**Authors:** Katrina S. McFerran, Helen Shoemark

**Affiliations:** 1 Melbourne Conservatorium of Music, The University of Melbourne, Australia; 2 Critical Care & Neurosciences, Murdoch Childrens Research Institute, Australia

**Keywords:** Students, vulnerable students, well-being, intellectual disability disorder, multiple disabilities, musical relationships, paradigmatic case study, video analysis, multiple perspectives

## Abstract

Students with profound intellectual disabilities disorders (IDDs) have the right to participate in educational opportunities that recognize their unique resources and needs, as do all children. Because of their specific communication challenges, positive relationships with attentive communication partners are critical for success. In fact, the power of positive relationships in schools is recognized to be connected to student well-being more broadly. This article examines the case of one young man with profound IDD and his relationship with his music therapist using a duo-ethnographic informed paradigmatic case study. Video analysis based on multi-voice perspectives is used to generate hermeneutic phenomenological findings to closely examine the relationship between a young man with profound IDD and a music therapist. The voices of four allied health researchers were also gathered to inform the authors’ construction of an informed commentary on the phenomenon. The results suggest that the essence lay in a combination of attentive, responsive and creative being with the other person over time. Four principles of musical engagement were identified in the video footage as critical to the meaningful relationships through music: the music therapist listens; the music therapist takes responsibility for structure; spontaneous initiation is sought from the young person; and the relationship is built over time. These concepts are contextualized within a discussion of student well-being that is underpinned by positive relationships and leads to students achieving their full potential within diverse school contexts.

Music therapy is a research-based discipline within which professionals are trained to work using the expressive and motivational qualities of music with individuals and groups to achieve non-musical outcomes relevant to their clinical needs. Music therapy practice in education is historically focused on students with a range of intellectual disabilities disorders (IDDs) (Carulla et al. 2011) and typically involves working towards goals with communication, social and motoric outcomes (Burge & Lester, 2001). In keeping with the dominant epistemology of Western school contexts, applied behavioral analysis has been the most common methodology for researching music therapy in education, with an emphasis on measuring the effect of music in terms of frequency and duration of observable, non-musical behaviors. This has not necessarily reflected the fullness of the lived experience for students since music therapists tend to practice from within person-centered models that emphasize empowerment and strengths even while striving for objective outcomes (McFerran & Shanahan, 2011). Lubet (2009) has noted a similar covert quality to the approach to music studies for people with disabilities.

In this article, we present a rich description of the process of engaging one young man with profound IDD in music therapy. The young man's profound disability included extensive functional limitation in his sensory, motor and intellect systems. The music therapist is a musician with university-level training in the design and facilitation of musical experiences specifically targeted towards the achievement of non-musical goals by participants. Within special schools that provide an education specifically for students with a range of disabilities, music therapists are often part of the well-being or allied health team, within which developmental goals are usually prioritized. Despite the general inclination within educational contexts to focus on observable behavior as the basis of evaluation, psychodynamic theory has continued to be an important theoretical influence within music therapy that leads to an emphasis on collaborative relationships established through, and in, music. While this relationship may result in the achievement of a range of observable well-being outcomes for young people, the lens in this case study is focused on inter-subjective rather than objective knowing. We argue that this knowledge is increasingly important in school contexts where relationships are increasingly recognized as critical to learning and development (Cahill, Shaw, Wyn, & Smith, 2004; Rickson, 2006).

Positive relationships within schools have been advocated from within a number of disciplines including positive psychology (Noble & McGrath, 2008), youth development (White & Wyn, 2004), educational leadership (Leithwood, Seashore, Anderson, & Wahlstrom, 2004) and of course educational pedagogy (Murray, 2002). Positive relationships within schools have frequently been identified as central to student well-being and resilience as measured directly (McGrath, 2005), as well as indirectly by impacting classroom management (Marzano, Marzano, & Pickering, 2003) and fostering strong school communities (Stipek, 2006). It could be said that there is large-scale agreement that caring relationships are necessary in order for students to thrive.

A body of research and descriptive reports document the perceived benefits of music therapy for young people with disabilities. This literature has been synthesized by authors using a range of strategies reflecting their paradigmatic stance—from meta-analyses with a focus on methodological requirements (Gold, Wigram, & Elefant, 2006), to descriptive analyses focused on categorization (Jellison, 2000), and deductive analyses focused on the elucidation of processes within music therapy (McFerran, Lee, Steele, & Bialocerkowski, 2009). There are limitations in the designs of many of the research studies according to “evidence-based” standards, since they have been conducted in real-world settings in which the number of participants is often too small and fully controlled conditions are not feasible.

Alongside the literature canvassed within reviews of the research, a narrative presence is also prominent in the literature, with interpretive case descriptions using theoretical frameworks from outside the discipline being common, with authors particularly adopting humanistic and psychodynamic lenses. Although the two traditions of research and description have co-existed within the music therapy literature for some decades, there is little overlap in their focus. Descriptive traditions have lacked sufficient rigor to be considered as case study research, and behavioral analysis has focused exclusively on outcome measurement of non-musical skill development with little acknowledgement of process. We believe this dualistic divide indicates a need for critical examination of the fundamental premises underpinning practice.

The achievement of student well-being within a musical relationship is central to music therapy practice in special education. However, it is only overtly emphasized in models where non-musical goals are discarded and creativity is prioritized (Aigen, 2005; Nordoff & Robbins, 1977). In North America and most parts of Australia, the school context demands a focus on individualized learning goals, and experimental researchers in this tradition usually attempt to address relationship by measuring social or communication outcomes. Nevertheless, this approach fails to capture the nature of engagement between the music therapist and student and it is this dynamic, and the subsequent well-being of the student, that is privileged in descriptions from music therapists in the field, as well as the families of students who are often advocates for music therapy.

The strong influence of interpretive traditions in the British and European context has led to an emphasis on psychodynamic theories of parent-infant interaction as a relevant way of understanding the nature of this relationship. Daniel Stern's (2010b) theory of affect attunement has been adopted by many music therapists in the Northern hemisphere because it provides an explanation for the inter-subjective matching that takes place when the therapist provides musical material that incorporates and develops participants’ musical contributions (Burns et al. 2002). However, the fit with Stern is not straightforward in the context of profound disabilities since he is clear that all dynamic interactional encounters rely on movement (Stern, 2010b, p. 89) and students with multiple disabilities are not usually perceived to have control over their body movement due to hypoxic brain injury or the severity of their neurodegenerative, genetic and metabolic disorders.

In addition to severe limitations in physical abilities, the communication skills of young people with profound IDDs are usually described as being at a *pre-verbal* level. This label has also been used in developmental psychology literature where the development of communication and social abilities through parent-infant interaction has been explored. We contend that this does not provide a useful focus for exploring communication with young people who will never become verbal, since it defines them by a skillset they will never achieve. A better framework may be the musical nature of the sympathetic interactions shared between parents and infants, that has been labeled by Stephen Malloch and Colwyn Trevarthen as “Communicative Musicality” (Malloch & Trevarthen, 2008). Communicative musicality is grounded in detailed analysis of the vocal and gestural synchrony shared between mothers and infants. It incorporates the work of Stern, as well as other significant contributors such as Ellen Dissanyake (2009) who have challenged developmental theories based on linguistic assumptions. Along with a range of musicologists, music psychologists and evolutionary theorists, Malloch and Trevarthen propose that all people are born with an innate ability for musicality that is fundamental to connectedness and unrelated to cognitive ability. Colwyn Trevarthen (1999) first described this as an “intrinsic motive pulse”, referring to the ways that we move in synchronous rhythm with one another. Related models such as coordinated timing (Jaffe, Beebe, Feldstein, Crown, & Jasnow, 2001) also rely on coordinated action as the basis of communication and assist in moving focus away from a trajectory privileging the development of speech and language, a change of direction that we argue is necessary for examining the well-being of young people with profound disabilities in schools.

In addition to significant cognitive and movement limitations, young people with profound IDD often have visual and hearing impairments, as well as epilepsy. This creates what is effectively an “averbal” state since the possibilities for achieving developmental milestones are severely impacted. Those who love and work with people with profound IDD know that this does not preclude the development of the individual's capacity for relationship, development of personality, lifestyle preferences and emotional development, all fundamental markers of well-being. However, it does suggest that cognitive and physical goals are secondary to the priorities for these young people and that meaningful relationships are an achievable and important focus, both within and beyond the school context.

The presence of students of all abilities within the mainstream school system has forced an expansion of the curriculum-focused agenda internationally. The concept of diversity rather than commonality is being used to describe the student population in Australian schools (Robinson & Jones-Diaz, 2006; White & Wyn, 2004) and even within that context, the needs of young people with profound IDD can be seen as distinct even from their special school peers. Although developmental goals will always be the focus of education, it is important to acknowledge the relevance of individual trajectories for each student. For this reason, a recent report in the UK advised that children with profound and multiple learning disabilities should not become the benchmark for students with special education needs (Kaestle, Halpern, & Brown, 2007), suggesting that a unique focus is necessary for these young people. While this thinking is embodied in the use of Individualized Education Plans within special education contexts internationally, the inclusion of this particular group of young people stretches any curricular expectations of educators. The diminishing scale of achievement only highlights the limitations of the educational framework to support each person's development across meaningful domains and thus an exploration of the previously unexplored dimension of well-being is timely.

Music therapists are often referred to work with students who have the most severe cognitive impairments. This may be because the inherently musical qualities of parent-infant relationships affords music therapists opportunities for unique roles in engaging the student with profound IDD and promoting the wider team's understanding of the child's idiosyncratic expressions of preference, joy and shared meaning. In this case study we attempt to contribute a systematically generated description of the process, by examining how music works as a vehicle for interaction and exploring the unique qualities of the relationship shared in music. The formal question that guided our investigation was “How does music stimulate the development of the relationship between a child with a profound disability disorder and a music therapist?”

## Methodology

A broadly hermeneutic phenomenological view informed our generation of a meaningful description within this study. van Manen's (1990) dynamic process of reflection, description, and interpretation is relevant here, contained within Flyvbjerg's (2006) concept of a paradigmatic case within which we considered the phenomenon being investigated to be representative of that which we sought to better understand. The specific focus for investigation was abductively generated by the authors based on our combined 25 years of working as music therapists with people who are non-verbal, both in special education (both authors), neonatology (Author 2) and pediatric palliative care (Author 1). Particularly within special education, both authors had experienced frustration with the failure of applied behavioral analysis to capture what we considered to be the essence of practice with young people who have profound IDD. We were inspired by Pierce's explanation of abductive process (described in Svennevig, 2001) as any observed facts that are generalized beyond the context in which they were first identified to essentially result in the creation of a hypothesis. The formula he uses to succinctly capture this process is:

The surprising fact, C, is observed;

But if A were true, C would be a matter of course,

Hence, there is reason to suspect that A is true.

(Peirce 1955, p. 151, cited in Svennevig, 2001)

In this case, we found it surprising that music therapists working with young people who have profound IDD actually do not focus on the development of communication and social skills in sessions, despite writing treatment plans that suggests this is our focus. But if this is because we spend more time focusing on fostering the development of the interpersonal relationship between ourselves and the young person, then our lack of focus on observable outcomes that signal skill acquisition is a matter of course. Hence, there was reason to suspect that music therapists implicitly focus on fostering the relationship between ourselves and young people with profound developmental delay as a strategy for enhancing the well-being of the student within the school context.

The hermeneutic phenomenological methodology was implemented through a duo-ethnographic process (Sawyer & Norris, 2004). Initial conversations between the authors over a period of 6 months were an attempt to dismantle a hegemonic stance by juxtaposing our stories, knowledge and practice about the experience of music therapy with young people with profound IDD. The iterative conversations were held both in person and through an online dialogue in which both voices were reflexively examined in relation to the long-held views generated by parallel clinical experiences. The narratives of each author manifested new realizations that were clarified and enhanced through reflexive dialogue about theoretical positions (Barry & O'Callaghan, 2008).

While both of us were respectful of the dominance of learning frameworks within the field of music therapy with children who have profound disabilities, both authors interpreted the lived experience through more psychotherapeutic lenses. As the first author noted, “I am listening, encouraging, providing a framework for the child to express themselves. I am not challenging, demanding, holding back, repeating—more learning strategies. It feels connected, powerful, creative, positive, enjoyable.” The second author was inspired to recall a quote by Abrams (2000), “The neonate's experience of music is not ultimately that of being impacted upon, or influenced by, a set of discrete sound stimuli—it is the experience of wholeness, or health, through sounds shared in a uniquely human way, through human contact”. While young people with profound disabilities are different from infants in many ways, in this case Author 2 felt that the music might provide a similarly whole and uniquely human form of synchrony for both the therapist and the young person.

The result of these long conversations was the decision to review the video footage and to privilege the notion of relationship as a focus. We invited multiple perspectives to assist in our attempts to transcend the existing theoretical stances and paradigms (van Manen, 2001). We selected a video sample that we felt adequately illustrated our focus from a collection of footage gathered as part of a larger investigation^1^ where traditional measures of communication skills had been used to determine whether or not the young person's communication abilities had observably improved as a result of 10 weeks of individual music therapy (McFerran & Stephenson, 2009). Using conservative statistical measures, no significant results were found in the larger study, although differences between the intervention (music) and the control condition (no music) could be visually observed in the graphed responses and videoed encounters. As a part of the behavioral analysis study, the first author had immersed herself in the video data over an extended period of time (in a process that might be argued as akin to extended participant observation within an ethnographic approach). She was therefore familiar with the video data and able to select 6 min of video from the 1900 min of data collected for the previous study. The sample was purposefully selected to represent a “good-enough” (Winnicott, 1964) moment that reflected the achievements of everyday practice and was not considered to represent a peak moment for the young person or therapist. We actively sought to avoid selecting material that represented an ideal case (Stake, 1995) and the paradigmatic case study design supported the selection of video data represented more general characteristics of interaction in question (Flyvbjerg, 2006).

The selection of only 6 min was a pragmatic decision, based on our desire to share the footage with other professionals to gather their descriptions, informed by different professional perspectives (see explanation below). Because we wanted to drill down into the data to generate an essence, we wanted to allow adequate time for extended discussion and felt that the selected footage was a satisfactory basis for that process. The limitations of that decision did have implications for how well the participants were able to understand the context, despite our attempts to overcome this hurdle by providing a descriptive contextual summary to assist the experts who volunteered to be involved.

## Soliciting expert perspectives

To challenge and complement our own perspectives, we engaged four allied health professionals with clinical or research experience in observation of non-verbal infant and child behaviors (including music therapy) to also micro-analyze the footage in an attempt to raise fresh language concepts that might stimulate new notions with which to discuss the phenomenon. Gathering a range of perspectives on a phenomenon in order to generate a rich description is a strategy used across a range of qualitative methodologies that are aligned with a constructivist paradigm. Egon Guba and Yvonna Lincoln (2005) describe researchers of this persuasion as “passionate participants” who are committed to multi-voice reconstructions (p. 194). Within music therapy, the gathering of voices has previously been used through hermeneutic panel studies (Loewy, 1995) and more recently, interdisciplinary inquiry (Bridgett & Cuevas, 2000) with the intention of micro-examining process. This approach has been considered illuminating because of the lack of established indigenous^2^ theory within the discipline of music therapy, combined with the unique challenges of describing interpersonal, musical experiences, and further combined with the tendency of music therapists to work in interdisciplinary teams. The values driving the data gathering methodology were of wanting to give voice to a young person whose profound physical and cognitive limitations make the objective discernment of voice extremely challenging. Relying on and valuing a range of subjective voices was one strategy we could use for listening closely and ultimately, striving to understand and meet the needs of young people with IDD in a more informed way.

The multi-voice perspectives came from four experts (all women) considered to have both theoretical and clinical expertise on interaction between professionals and young people operating at very early stages of communication development. The first participant was an international music therapy colleague who had completed a ground-breaking doctoral study with young women who have Rett Syndrome (Elefant & Wigram, 2005). The internal view of the phenomenon was considered to be represented by her perspective in the first instance, since Elefant's research revealed evidence supporting participants’ ability to communicate choices far beyond that which most professionals were willing to accept. Three allied health professionals from the state's leading pediatric hospital then provided the exterior perspectives and these women were selected because of their expertise in interpreting the behavior of children with neurological impairments. Finally, we also invited the music therapist whose work was being examined to contribute to the microanalysis. This perspective was sought in response to the questions raised by the four expert reviewers and she contributed a critical view that informed the subsequent analysis. Basic information about these expert participants is included in Table I.

**Table I:** Experts involved in microanalysis of video footage.

Expert	Field of expertise
1	Music therapist—research and practice with girls who have Rett Syndrome*
2	Physiotherapist—diagnosis team for cerebral palsy†
3	Occupational therapist—diagnosis team for cerebral palsy†
4	Speech pathologist—Paediatric Neuro-Rehabilitation Service†
5	Music therapist—therapist in the video footage*

* Interviewed by author 1.

† Interviewed by author 2.

## Data gathering protocol

The review of video footage and subsequent discussion took place in the quietest available room with each individual expert, although the Occupational Therapist and Physiotherapist watched together as they worked together as a team and wished to participate in the research together. Participants gave consent for their involvement in the study either verbally on the audio recording or by signing a written consent form. On the day of data collection, the experts were given instructions for the process. They were provided with information about Allan,^3^ a 16 year-old male whose physical diagnosis was Spastic Cerebral Palsy—Quadriplegia, epilepsy and a cortical vision impairment; as well as about the music therapist who was 10 years post university training; and their 2 year therapeutic relationship which had consisted primarily of individual sessions on a weekly basis. The experts were also given a context for the video footage, both in terms of what had happened in that session prior to the video excerpt and also the original purpose for collecting the footage (see Appendix 1), which had been to measure how long and how often the therapist and young person communicated in sessions, in what ways and with what degree of intentionality.

The experts were then given the opportunity to watch the video footage and were provided a form on which to write observations, interpretations and questions while they watched. The form was divided into 10-second intervals so that their comments could be clearly attached to a particular moment in the video footage. Immediately following the video review, the researcher and reviewer engaged in a discussion about the footage. In keeping with an epistemological stance that values specific co-constructed realities, there was some interchange of perceptions in these discussions. The researcher provided opportunities for each reviewer to lead the discussion and ask questions, thereby emphasizing the perspective of the other in order to gather new perspectives, while participating actively in the dialogue. The researcher answered questions to the best of her ability, represented her own perspective and actively sought more detail about the interviewee's perspectives. A review of video footage alone did not always afford the reviewer a clear understanding of subtle interplay, while discussion enabled implicit aspects of the expert observations to be raised and processed (Shoemark & Grocke, 2010). Throughout the duration of the study, the authors met regularly to discuss emerging findings and contemplate both anticipated and unexpected results. These discussions ultimately formed the basis for the emergence of four key principles, outlined below.

The authors generated a basic chronological description of the event on to which the interpretation of the interplay generated from the multi-voice descriptions was overlaid (van Manen, 1990). Finally the voice of the music therapist featured in the video footage was added to include the therapist's rationale and intention to produce the rich description (Denzin, 2001). The language of the musical interplay is drawn from the second author's *markers of interplay* study (Shoemark & Grocke, 2010). The first author prepared the rich description and the second author then both challenged and enhanced the interpretations by returning to the review transcriptions for direct language. Following the presentation of the rich description, we will discuss some of the issues that were elucidated through this process in relation to the research questions.

## Result: rich description

As we begin to watch Allan (young man) and Melissa (music therapist), Allan's head is up and his eyes are open and he vocalizes gently. Melissa immediately reciprocates with the same pitch in her voice. He vocalizes again, pitching a little higher, and she affirms his pitch and slides a little towards another note that creates a sense of musical anticipation for his response. From 2 years of sessions together, they both know that Allan can activate the nearby drum using a jelly-bean switch (6 cm round flat switch) located under his left elbow. He moves his elbow now and Melissa is ready to respond when he produces a sound. She provides a musical resolution by introducing a supportive harmonic framework on guitar—it suits the two notes Allan sang and the strong strumming pattern emulates the drum sounds, giving rhythmic energy to the shared music.

After the initial burst of energy Allan pauses. His body is mostly stilled and although his eyes continue to move it is in an involuntary fashion. To the naïve observer it looks as if his right elbow begins to move without purpose but this specific action would usually activate a large switch he often uses, which is broken today. Melissa entices further participation by continuing the energetic guitar strum and by repeating those initial vocalizations, but he again grows still. Melissa acknowledges the loss of momentum by vocalizing a concluding exhale-vocalization.

Melissa reduces the attack, tempo and volume on the guitar to provide a plucked style that is spacious in terms of rhythm and texture. She holds the musical thread with the original, mutually-created themes. She elongates the “space” at the end of the musical phrases knowing Allan may need time to respond. Allan seems disengaged, though his mouth and head move in the repetitive action typical of his athetoid cerebral palsy. He may be experiencing seizure activity. Melissa waits patiently while maintaining a gentle tonal pulse and offering more space until Allan vocalizes or plays. Each time he does so, she acknowledges his contribution with a gentle burst of texture and energy on the guitar before tapering off again to wait for his next expression. Throughout these moments, Melissa maintains the strum pattern but with modest attack and slowed tempo. Allan's continuous movement occasionally appears to border on purposeful expression, and Melissa pauses to allow any music he produces to be heard. When no sound emerges, Melissa moves smoothly into the next phrase, continuing to hold the musical thread for them both. It is predictable and structured, though not precisely repetitive.

After another minute, Allan re-emerges into the interplay and purposefully uses the jelly-bean switch to generate a loud bang from the drum. Melissa responds with a “Woo!” which emulates the volume and attack of the drum sound and Allan's apparent intention. While it might not be apparent to others, she knows that her high-energy musical response is likely to stimulate even more playing from him. Melissa comments, “Beautiful drumming”. Allan appears to be straining to use his voice and his quiet vocalizations prompt Melissa to put down her guitar and retrieve a microphone in order to amplify Allan's voice. To maintain continuity, she continues to talk to him as she sets up the technology. Her verbal commentary congratulates him “You are doing such a good job of drumming and singing”. With the microphone in range, the sound of Allan's vocal contributions can be heard more easily and he begins to vocalize and drum more consistently over these minutes.

Once Melissa resumes her position in front of Allan, she returns to strumming the opening upbeat sequence of punchy chords. Allan's head stabilizes and he smiles with intention and vocalizes his signature open vowel “ah” sound. Melissa takes turns vocalizing with him. Allan's constant dyskenetic movement stops and he smiles frequently. At times he turns his head and appears to listen intently to Melissa's music, while at other times he turns to vocalize into the microphone. He smiles as he turns his head away, apparently enjoying himself.

After a few minutes of interaction, Allan appears to lose energy and focus, almost as if he has withdrawn for a moment. Again Melissa acknowledges this reduced participation by reducing the volume of her playing and returning to the same gentle harmonic framework on the guitar. She softens the timbre of the now familiar chord structure, and her phrasing offers anticipatory space for Allan while moving on gently when he doesn't appear to be ready.

Allan yawns again and the music therapist mirrors the yawn vocally, offering a commentary on his tiredness “Woohoo, too much partying mister”. His body is still, and may be having an absence seizure as his eyes roll in circles for a little while. Melissa waits for him; she maintains the familiar chord sequence, using increased attack and volume towards the end of the phrase to entice his attention when he is available again. But as he remains absent, she returns to a gentle rendition of the chordal pattern. Melissa sings the original note contributed by Allan and he simultaneously re-emerges, smiling again and turning into the microphone as he vocalizes. Melissa emulates his modest energy. Whenever Allan looks as though he is about to make a sound with his mouth open and turning towards the microphone, she uses an abrupt stop on the top of a cadence to entice him to respond.

Allan's vocalizations are accompanied by smiles and he looks as though he is enjoying listening, and trying to move his body to play the drums through the jelly-bean switch. Melissa continues the supportive pattern of strumming and space for Allan. He is beginning to tire, and while there are three more vocalizations, they are spaced out by about 20 seconds in each case. He offers one further strike of the drum using the jelly bean switch under his elbow before the video ends.

## Analysis and discussion of the musical relationship

It was fascinating to consider the different perspectives generated by the multiple informants in this study, each of which has been incorporated into the rich description in some way. The intimate knowledge of the music therapist in the video provided a depth of explanation that the other music therapists could sense, but not articulate. The external allied health clinicians were often challenging in relation to the subtleties that could not be immediately understood—asking questions such as “why didn't she leave more space”, and “why is she being so constant in providing musical accompaniment”. Consideration of the questions that the allied health professionals asked, and ones that the external music therapists could not quite answer, revealed the unspoken dimensions of the relationships that underpin typical music therapy encounters such as these, and provided four key principles that we will use as the basis for the final discussion. One of the qualities that outsiders found difficult to perceive was that Allan's spontaneous contributions played a central role in directing the shape and structure of the encounter. Melissa's attentiveness to these subtle signs is equally critical, and she spends all of her time listening to and amplifying Allan's initiations. Between them a musical interaction takes places that is built on their collaborative efforts. In the following discussion, four principles of musical engagement will be used to discuss the relationship and the ensuing engagement in music—the deep listening of the music therapist, her willingness to take responsibility for structure, the emphasis placed on any spontaneous initiations made by the young man, and the fact that their ability to collaborate together has been built over time.

## The music therapist listens

Melissa's commitment to listening for Allan's contributions illustrates an essential principle of music therapy practice that is perhaps informed by the musical background required of music therapists. As musicians, auditory skills are highly developed over decades of playing and listening (Chin & Rickard, 2010; Strait, Kraus, & Ashley, 2010). There is a substantial literature on the music perception of musicians compared to non-musicians that confirms this statement (i.e., Thomsen & Rekve, 2006), although there are controversies about whether people with a genetic predisposition for music are attracted to learning an instrument (i.e., their neurological propensity for enhanced music perception is pre-existing) or brain plasticity is influenced by the years of practice that lead to an improved neurological capacity for hearing. This debate is part of a larger nature versus nurture discussion. For the purposes of this study, it seems feasible that the music therapists’ perception of sounds created by participants may be better attuned than other professionals.

This neurological basis is also emphasized in professional training. Music therapists develop musical listening skills as a fundamental competency. The importance of listening is attached theoretically to humanistic theory, based on Carl Roger's emphasis on the importance of empathic listening (Rogers, 1951). This has been developed in more contemporary music therapy theory as one of the central values of the musical encounter. “Musicing with other people involves listening intently to them and responding in a way that reflects that listening” (Aigen, 2005, p. 83). Of particular relevance to this special education context is the art of “listening-in-playing” (Ansdell, 1995) that was also identified in an international study of improvisation in music therapy (McFerran & Wigram, 2002).

The child's right to be heard has been clearly stated within the UN Convention on the Rights of the Child (Hart, 2002). Article 12 emphasizes that young people's views be not only expressed, but also to be given due weight within a developmentally understood context. For most children, schools are an important site for this doctrine to be actioned and yet the hierarchical nature of the system can place limits on the degree to which this happens. Nonetheless, the importance of being listened to has received some attention within the school context and pupil voice is a critical dimension of the Every Child Matters agenda in the UK (Cheminais, 2008; Whitty & Wisby, 2007). Policy is supported by research, with a meta-analysis of over 100 studies, which revealed that the quality of the student-teacher relationship was positively related to better classroom management, and that teacher listening was one of qualities required along with being empathic and interested (Noble & McGrath, 2008).

However, listening in the context of a child with IDD is not always easily understood and Melissa's attentiveness to Allan's contributions was not immediately apparent to the reviewers who questioned whether she left enough space for his responses.

Physiotherapist: I thought she could have given him more. I just wanted her to be a bit quieter, a second more, and just let him complete his head movement and see what he was going to do. But she was listening. I was watching …

This challenge from the reviewers helped us to articulate the first principle, since it was apparent to the music therapists that Melissa was listening attentively, whilst playing. While turn-taking is seen as an ultimate achievement of shared verbal encounters, music offers for a unique possibility of “speaking at once”. Theory suggests that the human ability to play together in time was fundamental to our evolution (McNeil, 1995), allowing us to build communities and to share joyous experiences that reflect the social rules of society (Cross, 2008). Although it may have been fundamental, contemporary society reveres verbal communication and this is the dominant lens through which communication is understood. There is no existing measure for simultaneous communication, and this has been problematic for previous analyses of musical encounters between music therapists and children with profound and multiple disabilities (McFerran & Stephenson, 2009).

## The music therapist takes responsibility for structure

The ongoing musical framework provided by the music therapist during improvisations is designed to “create a musical structure that can allow (and inspire) the development of more expressive and creative playing by the client” (Wigram, 2004, p. 118). This can be explained using theoretical understandings of parent-infant encounters because of the similarities in purpose (fostering development) as well as the form of the encounter (Tiggemann & Slater, 2004). The form of parent-infant encounters has been described as inherently musical, with pulse, quality and narrative being identified as the three defining parameters (Malloch & Trevarthen, 2008). Pulse is the process within which parent and infant coordinate their actions (rather than turn-take in a strict sense). The expressive qualities of the encounter are considered to be multimodal, with an interplay between vocal sounds, body movements and facial expressions marking the dynamic interplay (Shoemark & Grocke, 2010), referred to as vitality forms by Stern (Burns et al. 2002). The pulse and quality come together in a narrative that draws on the shared history of the two participants as they share time in the present moment. This understanding of musical and averbal communication helps to explain why Melissa listened and played at the same time, while waiting for, and encouraging Allan to participate.

Interviewer 2: We obviously make an assumption about what she's doing, but it's difficult to understand what's going on. (Occupational Therapist): Yes, see this is where I wrote “Why did she change the pace, volume, vocalization? Interviewer 2: In a certain period of time she would expect him to respond and engage. So she's just shifting the music slightly to give him that little bit of encouragement just in case he's able to continue, to come back in. You know, clients will disengage a little but if you leave it then they drop out of it completely so I just wondered if it's that, just trying to stop him from dropping out completely. (Occupational therapist): *That's* the skill of the therapist, the absolute skill, about picking, “Have you just disengaged? Are you engaged and just trying to respond, do I need to wait?”. I would expect that that's a really important skill for a music therapist working with such a severely disabled child when the response was not always consistent.

This conversation illustrates how it was the implicit knowing of the music therapist that needed to be articulated in the principles generated by this investigation. The need to provide structures for learning is integral to the special education sector, where the concept of scaffolding activities is highly relevant (Hallahan, Kauffman, & Pullen, 2000). Although we argue that music therapists are structuring musical participation in service to the building of relationship rather than in the development of the kinds of skill sets that are sought in the educational context, there is an overlap between communication and relationship. As described above, the unique structures identified with music happen in a nuanced way over time, making them even more subtle for the non-musician to discern, and it is possibly for this reason that the arts are seen to make an important contribution to the individual child flourishing within the school context (Stokes, 2003).

Nygaard-Pederson's (2007) delineation of the “listening attitude” can be understood as drawing together the principle of listening (described first) and encouraging spontaneous initiation (described next) through the provision of musical frames and structures. Pederson draws on her experience with adults who have severe mental illness and describes how the music therapist's careful listening fosters initiation, because the listening happens actively. “No matter how rudimentary or fragmentary the music might sound … (I) simultaneously create a musical frame that resonates with what I hear” (p. 31). The musical encounter does not cease once Allan becomes an active agent in the relationship and he relies on Melissa's recollection of their existing relationship, both in the moment and over the years, to provide ongoing possibilities that extend his capacity to be heard.

## Spontaneous initiation is sought from the young person

Spontaneous initiation has been increasingly noted in the music therapy and special education literature and Melissa describes working towards a state of optimal arousal that would support Alan's participation. “So I guess there I'm just using that music as a bit of a call to see if he still wants to continue on with that same song. Otherwise I guess I'd move on to something different, either a new song or more space, but … coz that sort of motif seemed to be working before, I just wanted to see if he was going to rejoin again or something different. And he just, like, sort of smiled in his eyes”. Although this has not been measured in the field of music therapy and IDD, increases in spontaneous initiation have been captured in studies of music therapy with children who have less profound cognitive deficits because these encounters follow the rules of conventional verbal interactions, i.e., they have less musical frameworking and more musical turn-taking. Kim used a randomized controlled trial to compare the communication behaviors of young children with autism in music therapy and a play condition, and revealed that initiation was one of the significant differences (Kim, Wigram, & Gold, 2008). Similarly, a study of music therapy with deaf children following cochlear implants identified that spontaneous interaction, particularly in relation to turn-taking, was the most significant improvement between the control and the intervention condition (Kelvin, Goodyer, Teasedale, & Brechin, 1999).

Because of the severity of Alan's IDD, this focus on spontaneous initiation was not obvious to the reviewers in this study, who felt that the musical frameworking and simultaneous communication left insufficient space for his contribution.

Occupational Therapist: I'm confused sometimes when she jumped in and she didn't jump in. Like, she would play a bit and then wait for a response and there were multiple different responses. Was it a smile, was it a vocalization, was it a switching kind of thing? And then sometimes when she didn't get a response and she would move on and then also it was interesting because sometimes when she got a response, sometimes she would “Yay, that's fantastic, yay”.

The effort exerted by Allan illustrates how challenging this expectation is for people who have profound IDD. Vision impairments limit perception, and the impact of epileptic seizures was queried by the participants in our study, particularly the possibility of “absence” seizures that result in moments of unavailability. While the participants in the study spent considerable effort identifying these limitations and questioning the likelihood of intentionality in Allan's playing, the music therapist was focused on the possibilities for participation on the day, drawing on her knowledge of how to encourage Allan rather than focusing on his limitations. The phrase “maximizing islands of potential” has been used to describe this tendency in music therapy encounters, based on analysis of the dyadic encounter between music therapist and child in pediatric neurorehabilitation (Bower & Shoemark, 2009). The purpose is to counter the learned helplessness (Took & Weiss, 1994), which can result when a person is considered to have little potential for initiative and has to borrow the capabilities of their partner to exert agency.

Within the special education context, being able to initiate communication is seen as important for students with IDD because it enhances the possibilities for control over their environment (Sigafoos, Butterfield, & Arthur-Kelly, 2006), which is critical for well-being. A literature review of 20 years of evidence supporting the development of communication interventions with students who have IDD identified that, despite gathering evidence of improvement across a vast range of communication skills, initiation and spontaneous communication was impossible to measure because of the challenges of inter-rater reliability with this group (Snell et al. 2010). The practical challenges of acquiring the capacity for spontaneous initiation are also noted by Carter (2003), whose research showed that spontaneous communication had the least success in being understood by the communication partner, and who emphasized the importance of the partner being able to wait. The possibilities offered by music therapy are therefore somewhat unique in this context.

## The relationship is built over time

Another essential principle of the encounter that was difficult for the reviewers to perceive in the video footage was the context of the 2-year relationship between Melissa and Allan and the inclusion of Melissa's voice in the research dialogue illustrated some of the shared meanings that had been missing to that point. The prior existence of a “jelly-bean” switch under the right elbow, broken on the day of the recording, provides a concrete illustration of this local knowledge. An understanding of Allan's energy level and availability for interaction in each phase was also inherent in Melissa's insights as she explained aspects of interpersonal timing:

Music Therapist: He would ebb and flow all the time, so he just needs a breather for a second … so sometimes I just do this underneath [play plucked chords on guitar]. I think that's different from speech, because, if I was just talking I'd feel, either not talking and there was a big gap, or, I was talking and that's not what he needed. He just needs something holding, just something really gentle, holding underneath, which you can do with something like strumming guitar.

In music therapy with medically fragile newborn infants, Shoemark noted that while sick infants can realize their musical potential, the therapist must account for the infant's complex daily reality in crafting a successful experience (Shoemark, 2006). Melissa had assembled and tested a range of knowledge about Allan that validated her construction of this specific engagement:

Music Therapist: Initially I was trying to engage him, but I think that awareness and that history that we had together is that I know his energy levels go up and down. So I got a baseline from when he came in [to the music room] and some days he would come in and he could hardly do anything, and then the sessions would be totally different.

Such qualities are inherent in the parent-infant encounter and drawing on his research with these dyads, Stern (2010a) proposes that all people have lifelong *vitality forms*, which are the idiosyncratic and non-verbal features of our interactions with others. Our regular social partners come to know our melodies, stress, volume modulation and vocal tension (p.122) and then attunement occurs through the creation of shared meaning.

Stern's concept of affect attunement has been explicitly linked to the achievement of therapeutic change in psychotherapy through research conducted by the Boston Change Group (Trerotoli, Soldano, Serio, & Moretti, 2005). A model of mutual regulation between two persons is hypothesized as underpinning why positive relationships are linked to therapeutic outcomes. Stern's elucidation of “the mother's attempt to share the infant's intersubjective experience” (Stern, 2010a, p. 114) by imitating the dynamic features of the child's communication but not simply copying his/her actions is evident in the phenomenological description generated in this study. An alternative frame for understanding the interaction between Melissa and Allan is cross-modal reciprocity and emulation (Shoemark & Grocke, 2010). When Allan moves, Melissa reciprocates, using pitch and other musical elements in combination, or she changes her guitar accompaniment to emulate his vocalizations, leading out with more energy in her musical response (Shoemark & Grocke, 2010).

As emphasized by most theories, these encounters take place in time and draw on knowledge that has been accrued over time. Holck's (2007) microanalysis of music therapy sessions with adolescents who have profound disabilities details the interaction themes that emerge over time. In her study, the musical themes consisted of simple and repeated musical phrases that were often initiated by the young person and then developed continuously as the weeks passed (Holck, 2004, p. 8). The themes were thought to generate expectations between the therapist and child regarding the shared music making, and these made it easier for both social partners to understand the interaction as meaningful and to keep the threads of interaction going over time. The intentionality perceived by the music therapist is informed by this context and is not necessarily visible to objective observers, such as the reviewers in this study, nor is the degree of consistency in responses perceivable through the analysis of a single encounter.

The nature of most school contexts embodies this knowing about the importance of building relationship over time, and in special education it is common for teachers to work closely with the same students for at least 1 year at a time. Managing the number of years that teachers remain with students in order to foster relationship development has long been acknowledged (Miles & Darling-Hammond, 1998), but is seldom documented in the literature. Despite this, the increasing focus on the importance of relationships in schools, combined with the challenges faced by students with IDD, leads easily into recognition of how a longer-term relationship can be valuable.

## Conclusion

In this research, we attempted to drill more deeply into the process of developing the kinds of relationships that we perceive as existing beneath the social and communicative encounters that are observable in music therapy with young people who have profound IDD. Our abductive process was based on the surprising fact that improvements in the communication skills of young people with profound IDD were not observable using conventional communication measures focused on turn-taking (McFerran & Stephenson, 2009), despite the agreed perception of clinicians and families that music therapy is a valuable communication experience for young people. We proposed that music therapists spend less time targeting the development of observable pre-verbal communication skills and more time fostering the experienced relationship between themselves and young people and that they do so for a different therapeutic purpose, as yet unarticulated in the literature. Four key principles of musical engagement were identified through consideration of questions arising from the solicitation of multiple perspectives on the relationship, as viewed through a paradigmatic case illustrated by a 6-min video sample. The principles are that:

- The music therapist listens
- The music therapist takes responsibility for structure
- Spontaneous initiation is sought from the young person, and
- The relationship is built over time.

These principles were considered in light of both music therapy research and practice from school-based contexts. In the case of young people who have profound IDD, amplifying their capacity and relating to them in a meaningful way may not lead to the kind of development that we anticipate for infants as they grow because of the significant cognitive and physical limitations that mark their lives. Further research is needed to explore more innovative understandings of the relationship in light of emerging research capacities.

Occupational Therapist: “If you really enjoy doing something, you get endorphins. So how does it make him feel to have a great session? It probably makes him feel good for a few hours. Surely giving him a happy experience of pleasure, giving him a release of serotonin or whatever …”, and in response,

Physiotherapist: “Surely music is fundamental for every human being.”

We propose that the fundamental importance of sharing these relationships is a human right that young people deserve. Being heard and acknowledged within supportive relationships in the school context is potentially even more important for students who have profound IDD, and it is clear that music therapists are able to manifest such relationships over time and draw upon the unique potential of music. How this is transferred from within the music therapy session to other parts of the students life then becomes a critical challenge for the discipline, and has been addressed in emerging models that embrace ecological theoretical perspectives (McFerran, 2012). By actively striving to generalize positive experiences of relationships beyond the therapy room, music therapists will be able to make a more significant contribution to student well-being while simultaneously acknowledging the strong emphasis they place on the development of relationships with students who have profound IDD.

## Footnotes

1 Ethics approval was granted for this sub-project: University Of Melbourne DHEAG Approval #0931563.1.

2 The term indigenous has been appropriated by Kenneth Aigen (1991) to describe theory that is unique to the discipline of music therapy and not imported from psychology.

3 Please note that this is a pseudonym.

## Appendix 1

### Information provided to experts prior to video analysis

***Duration: 6 minutes***

For the 10 min prior to this, the music therapist has been singing and playing the guitar with the young man. She has used a greeting song that they sing each week, followed by another song that is more upbeat and familiar to him. Just prior to this segment she has offered the drum for the young person to play, and has set it up to be triggered by a large jelly-bean switch under his left elbow. This has taken some time to position, but he has started switching immediately. The clip begins as she slides into place and begins to play the guitar.

***Participants***

Music Therapist: She has approximately 10 years of experience and works from a broadly humanistic orientation. At time of session had worked with young man for 2 years.

Client / Young man: Attends music therapy as part of his school program. Attends the school because of disabilities caused by Spastic Cerebral Palsy (Quadriplegia), epilepsy and a cortical vision impairment. In this footage he uses the switch under his left elbow, which is attached to a drum (out of sight). He also vocalizes.

***Prior analysis of this footage***

The footage is taken from a series of data collected for Stage 1 Analysis. The focus of this analysis was on observing pre-intentional and early intentional communication: specifically Initiations and Responses. This included three facets:

1. How often each communicative partner appeared to initiate or respond—looking for turn-taking within this framework.
2. How the child appeared to do this—body movements; gestures without contact; gestures with contact; vocalization; self-injurious behavior; or other aversive behavior.
  How the therapist did this—Provide opportunities to: attend to RMT or an event; choose between options; request help or more; make a comment; answer a question; reject or protest; greet/farewell.
3. What the (subjectively) perceived intention was: Reflexive; Goal Directed; or Intentionally Communicative.
